# Repellents against *Aedes aegypti* bites: synthetic and natural origins

**DOI:** 10.3389/finsc.2024.1510857

**Published:** 2025-01-22

**Authors:** Melissa Noguera-Gahona, Cindy Peña-Moreno, Natalia Quiñones-Sobarzo, Caroline Weinstein-Oppenheimer, María Guerra-Zúñiga, Ximena Collao-Ferrada

**Affiliations:** ^1^ Virología, Laboratorio Clínico, Facultad de Medicina, Universidad de Valparaíso, Viña del mar, Chile; ^2^ Facultad de Farmacia, Universidad de Valparaíso, Valparaíso, Chile; ^3^ Centro de Investigación, Desarrollo e Innovación en Productos Bioactivos, Universidad de Valparaíso, Valparaíso, Chile; ^4^ Departamento de Salud Pública, Facultad de Medicina, Universidad de Valparaíso, Viña del mar, Chile; ^5^ Centro Interdisciplinario de Investigación Biomédica e Ingeniería para la salud, Universidad de Valparaíso, Viña del mar, Chile

**Keywords:** repellent activity, synthetic repellents, natural repellents, arboviruses emergence, dengue prevention strategy

## Abstract

Dengue fever, transmitted by mosquitoes of the *Aedes* genus, particularly *Aedes aegypti*, has emerged as a global health issue. With the expansion of this mosquito to new geographical areas, driven by factors such as climate change, the need for preventive measures like using insect repellents has become critical. The present review explores the current state of the art on topical mosquito repellents, both synthetic and natural, used globally, especially in regions where dengue is endemic. Among synthetic repellents, DEET is the most widely used, supported by investigations demonstrating its efficacy and safety, although concerns about its toxicity exist in exceptional cases. Other compounds, such as picaridin, IR3535, and PMD are also common and offer alternatives with variable safety and efficacy profiles. Natural repellents, such as essential oils of citronella, lemon eucalyptus, and clove, have proven effective against *Aedes aegypti*. However, they present challenges due to rapid volatilization and the limited duration of their protective effect. To address these issues, combinations of essential oils and synthetic compounds have been proposed to improve efficacy and safety. Finally, the review highlights the complexity and the challenges of developing new repellents, including the high costs and lengthy timelines for commercialization, as well as the importance of continued research to improve the efficacy and safety of these products.

## Introduction

*Aedes aegypti*, a member of the *Culicidae* family, is an important arbovirus vector responsible for millions of cases of dengue, Zika, yellow fever, and chikungunya annually (1). Found mostly in tropical and subtropical regions, it shows remarkable adaptability, allowing it to expand to new territories (2). Female *Aedes aegypti* mosquitoes feed on the blood of infected hosts and acquire arboviruses that first establish themselves in the intestinal epithelium before spreading systemically throughout the mosquito’s body (3). Once the virus reaches the salivary glands, the mosquito can transmit it to new hosts during subsequent bites (4). After feeding, females lay eggs along the edges of standing water sources, where the eggs can remain dormant for months and hatch when conditions become favorable (5). One of the primary arboviruses transmitted by *Aedes aegypti* is the dengue virus (DENV).

DENV is part of the *Flavivirus* genus and *Flaviviridae* family (6). It is an enveloped, positive-sense RNA virus with four serotypes (DENV-1 to DENV-4), classified by shared antigens (7, 8). The infected person may develop dengue fever 4–7 days after being bitten by an infected mosquito, and the symptoms may last a week or more (9, 10).

Dengue fever can be life-threatening, especially in children, and has become a significant global health concern (11). Its impact is particularly severe in tropical and subtropical regions, but the disease has also spread to previously unaffected areas, now placing over 40% of the world’s population at risk (12). Recent estimates indicate that more than half of the global population (approximately 3.6 billion people across 129 countries) reside in regions at risk for dengue infection. In 2023, the World Health Organization (WHO) reported a staggering 10-fold increase in dengue cases, rising from 500,000 in 2000 to 5.2 million in 2019 (13).

Dengue transmission occurs in the Eastern Mediterranean, the Americas, Southeast Asia, the Western Pacific, and Africa, with new cases spreading to nonendemic areas in the USA, Europe, and the Americas (10, 14). Asia reports the highest incidence, followed by the tropical regions of the Americas. In Africa, dengue rates remain unclear due to misidentification with malaria, although a steady increase in cases has been observed over the past 40 years, as well as in islands in the Indian and Pacific oceans (10). The Americas have seen a dramatic rise in cases, with over seven million reported by April 2024, surpassing the previous peak of 4.6 million cases in 2023, underscoring the rapid escalation of this global health issue (14).

Several factors, including climate change, deforestation, and globalization, have contributed to the expanded spread of *Aedes aegypti* in recent years, creating favorable environmental conditions that allow the mosquito to colonize regions where it was previously absent. Consequently, entomological monitoring and control measures are crucial to detect and manage mosquito populations, minimizing the risk of outbreaks or epidemics. Moreover, as *Aedes aegypti* expands into new areas, promoting individual protective measures against mosquito bites becomes essential. Topical insect repellents, widely recommended by health authorities, are among the most effective strategies for preventing mosquito-borne diseases (15). The WHO specifically recommends the use of mosquito repellents containing DEET, picaridin, or ethyl 3-[acetyl(butyl)amino]-propanoate (IR3535) as effective options (16).

In countries with high prevalence rates of mosquito-borne diseases, a wide range of commercially registered repellents exists; despite this, few active ingredients are used in these formulations. Conversely, in regions with low dengue prevalence, the availability and development of repellent products are limited, and knowledge of their characteristics remains insufficient. Therefore, the expanding distribution of *Aedes aegypti* justifies a review of the current status of these products and an understanding of their characteristics, from their origins to their application.

This review aims to provide an overview of the characteristics, efficacy, and safety of topical mosquito repellents, both synthetic and natural, based on the experiences of countries where prevention measures are well documented, with particular attention to their significance in regions recently affected by the presence of *Aedes aegypti*.

## General information on repellents

Countries that produce repellents commercially, such as the USA, Brazil, India, and Australia, regulate and assure their quality and efficacy through national regulatory agencies, including the Environmental Protection Agency (EPA) in the USA, Agência Nacional de Vigilância Sanitária (ANVISA) in Brazil, the Central Drugs Standard Control Organization (CDSCO) in India, and the Australian Pesticides and Veterinary Medicines Authority (APVMA) in Australia. These agencies oversee product registration and ensure compliance with safety and efficacy standards (15–20). Registered repellents are labeled with information about their active ingredients, concentrations, safety precautions, and recommended reapplication intervals, reflecting the variation in protective duration among different active ingredients.

In Brazil, synthetic repellents are produced on an industrial scale, with DEET, IR3535, and picaridin being the most commonly used. Additionally, some plant-derived extracts and essential oils are also incorporated into repellent formulations (21). Similarly, in Australia, DEET, picaridin, *p*-menthane-3,8-diol (PMD), and various plant-based products, such as tea tree oil, eucalyptus, and citronella, are frequently used (15). In the USA, the most common EPA-registered repellent ingredients available on the market include DEET, picaridin, IR3535, PMD, and lemon eucalyptus oil (which contains PMD but is regulated separately) (22). In India, diethyl phenyl acetamide (DEPA) is commonly used as an alternative repellent, alongside DEET, picaridin, IR3535, and PMD (23).

## Development of synthetic products

To begin with, the most widely recommended synthetic repellent at the international level is DEET. For over 50 years, it has been the basis for numerous and varied commercially registered formulations on the market, containing concentrations of 10% to 80% DEET. There is considerable scientific support for its safety and effectiveness (15). Conversely, there are published reports describing severe toxic properties that could affect adults, and especially young children, such as dermatitis, allergic reactions, and neurotoxicity (24). However, the risk of developing these effects is considered minimal with typical use. Nevertheless, it is recommended that DEET be used at the lowest effective dose possible (between 20% and 30%) (25).

In addition, picaridin has also been shown to provide protection at concentrations of 10%–20%, comparable to DEET, for up to 5 h against *Aedes aegypti* bites (19). Formulations of this compound vary in concentration from 9% to 20% (15). Together with DEET, it has been widely recommended by authorities internationally (19). Picaridin-based formulations are considered cosmetically more suitable for use (15), and although its safety has not been investigated to the same extent as DEET, it is considered a safe product (26). DEET and picaridin are considered safe for use by pregnant and breastfeeding women (19). However, safety recommendations for use in young children are included in the registered products due to a lack of safety data.

Alternatively, in some investigations, the compound DEPA, used in India, has been shown to be suitable for topical use, with similar efficacy to DEET and mild to moderate toxicity for dermal use (27, 28).

Another compound that provides protection comparable to DEET is IR3535; however, it requires frequent reapplication, every 3–8 h. Although it is considered a safe ingredient for use as a repellent, substances derived from this compound, which exhibit mutagenic and carcinogenic properties, have been found in the chlorinated water of public swimming pools (29).

Finally, formulations that contain PMD, a component of the essential oil of *eucalyptus* spp., are available and recommended for their effectiveness, with their repellent properties clearly established (30). However, this product must not be confused with eucalyptus-based essential oil formulations, as these have not demonstrated an equivalent period of protection against mosquito bites, such as PMD. PMD is considered a by-product of the lemon eucalyptus hydrodistillation process and not the essential oil itself (15). Laboratory studies have demonstrated that a 30% PMD formulation provides a duration of protection against mosquito bites comparable to that of repellents based on low doses of DEET (5%–10%) with picaridin (30). In laboratory studies with animals, PMD has proven to be safe, although it may cause eye irritation, so caution is advised for use on young children (31).

## Development of natural products

Due to their safety and efficacy, as well as being environmentally friendly, biodegradable, easily accessible, and lower in cost, interest has grown in natural repellents as safe and effective alternatives. Currently, numerous studies in the literature describe plant derivatives with repellent properties, as plants have historically been a source of insect control agents, especially against *Aedes aegypti* (32–34). Although these products are not exempt from some adverse effects on the skin (35), these problems can be overcome by using certain topical formulations, such as solid lipid nanoparticles (36), creams (37), emulsions (38), and gels (39).

The effectiveness of protection provided by synthetic products and numerous plant derivatives against mosquitoes, such as *Aedes aegypti*, *Anopheles stephensi*, and *Culex quinquefasciatus*, has been widely evaluated. In general, *Aedes aegypti* is the most difficult species to repel. However, several essential oils that have proven effective against this species have been identified (38, 40).

Among the most relevant are the essential oils of citronella, lemon eucalyptus, and clove, among others (38, 41, 42). These oils have been used as mosquito repellents for over a century, and different authors report them as the most widely used natural repellents in the world today. Their main characteristics are shown in **Table 1**.

**Table 1:** Essential oils with repellent activity commonly used against *Aedes aegypti* mosquitoes, their main active components, and bibliographic references.

Name of essential oil	Country of origin	Main active components	Bibliographic references
Lemon eucalyptus oil (*Corymbia citriodora*)	Australia	*p*-Menthane-3,8-diol (PMD), citronellal, citronellol	(44, 47–49)
Citronella oil (*Cymbopogon winterianus*)	Indonesia, Sri Lanka, India	Citronellal, geraniol, citronellol	(44, 50, 51)
Lavender oil (*Lavender angustifolia*)	France, Bulgaria, Spain	Linalool, linalyl acetate, 1,8-cineole	(44, 52)
Peppermint oil (*Mentha piperita*)	USA, India	Isomenthol, p-menthone	(44, 52)
Basil oil (*Ocimum basilicum*)	Italy, India	Linalool, methyl eugenol, 1,8-cineole, limonene	(44, 53)
Lemon oil (*Citrus limon*)	Italy, Spain	Limonene, beta-pinene, gama-terpinene	(44, 52, 53)
Clove oil (*Syzygium aromaticum*)	Indonesia, Madagascar	Eugenyl acetate, eugenol, beta-caryophyllene	(44, 48, 53)
Thyme oil (*Thymus vulgaris*)	Spain, Morocco	Thymol, *p*-cymene, geraniol	(44, 53, 54)
Geranium oil (*Pelargonium graveolens*)	Egypt, China, Morocco	Citronellol, geraniol	(38, 44, 55)

A variety of other essential oils that have demonstrated activity against *Aedes aegypti* include, for example: mugwort (*Artemisia vulgaris*), bay laurel (*Laurus nobilis*), camphor (*Cinnamomum camphora*), cassia (*C. cassia*), cedar (*Cupressus L., Juniperus L.*), chamomile (*Chamaemelum nobile*), catnip (Nepeta cataria), lemongrass (*Cymbopogon citratus*), cinnamon (*Cinnamomum zeylanicum, C. verum*), fennel (*Foeniculum vulgare*), aromatic litsea (*Litsea cubeba*), violet (*Viola odorata*), galbanum (*Ferula galbaniflua*), jasmine (*Jasminum grandiflorum*), juniper (*Juniperus communis*), myrtle (*Myrtus communis*), black pepper (*Piper nigrum*), rosemary (*Rosmarinus officinalis*), sage (*Salvia sclarea*), sandalwood (*Santalum álbum*, *Santalum* spp.), marigold (*Tagetes minuta*), verbena (*Lippia triphylla*), garlic (Allium sativum), and neem (*Azadirachta indica A. Juss*) (43, 44). Other combinations can also be found, such as citronella with vanillin, geranium with citronella, turmeric (*Curcuma longa*) with vanillin, or basil (*Ocimum americanum*) with vanillin (44). It has been described that the fixative material vanillin increases the magnitude and duration of the repellent effect of both DEET and plant essential oils (45).

## Bioactive components in essential oils

**Figure 1:**
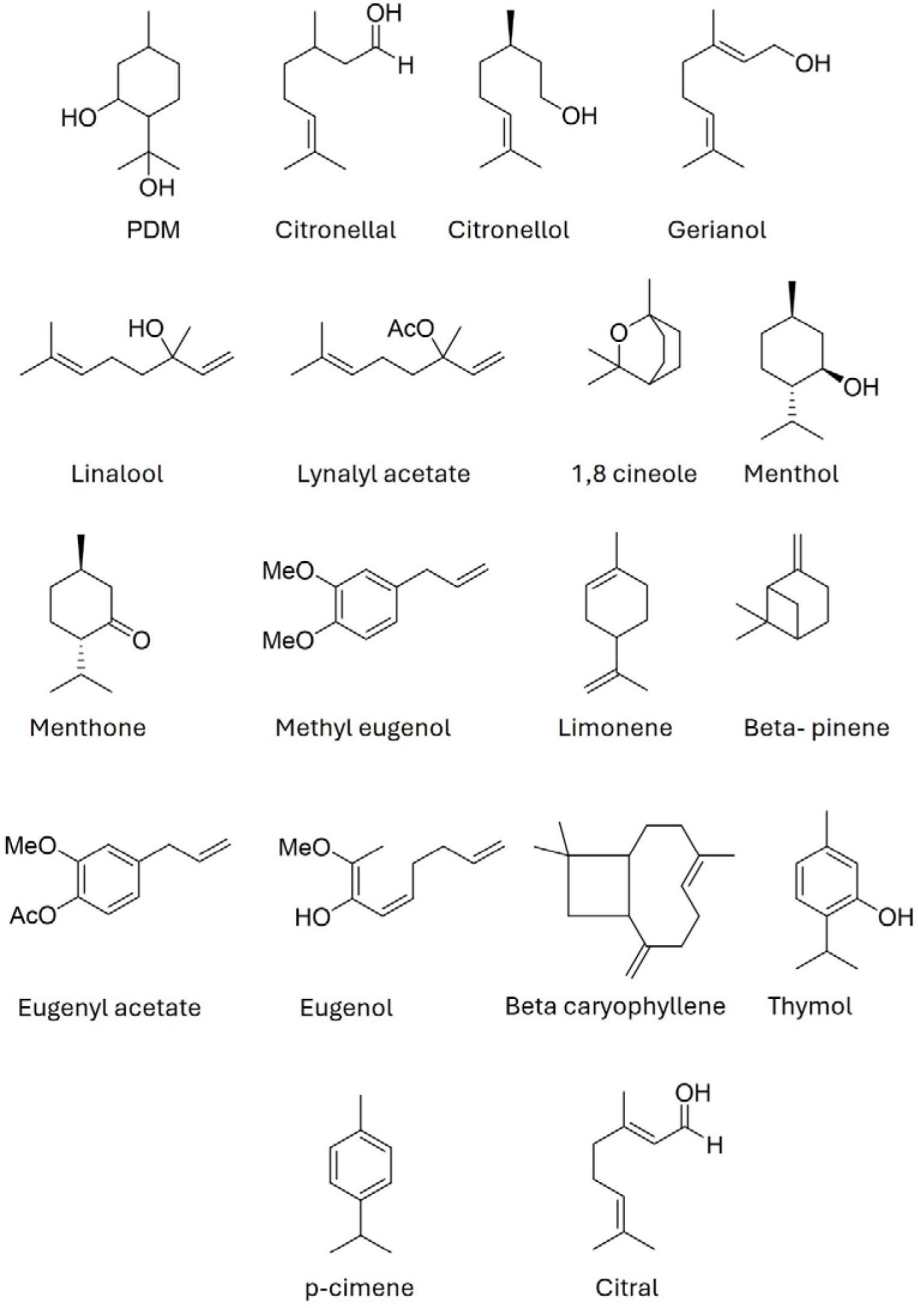
Structures of some components of essential oils.

To understand the mechanisms by which essential oils present repellent activity and advance the development of products with this activity, it is essential to characterize the bioactive compounds present in these extracts. Essential oils are responsible for the aroma of plants, and most of the molecules that compose them are volatile. The variety of molecules present in an essential oil can range from a few to several hundred, with this also varying depending on the growing area and environmental conditions (46). The components of essential oils can belong to different classes of molecules, such as terpenes, alcohols, aldehydes, ketones, and phenols, among others. However, the main compounds in essential oils can be classified into two main groups: terpenes (mainly monoterpenes) and phenylpropanoids. In this sense, terpenes represent over 90% of the components of essential oils (56), and the repellent activity of essential oils has been associated with the presence of monoterpenes and sesquiterpenes (45). Some monoterpenes identified from essential oils with insect-repellent activity are shown in **Figure 1**.

These compounds can act on insects through several mechanisms, including interactions between essential oils and multiple different olfactory receptor systems, including odorant receptors in mosquitoes (38). It is thought that mosquito repellency is due to synergistic interactions of the chemical components present in the essential oils, which would help to enhance their effectiveness.

Therefore, since a variety of repellent products have been developed in the last decades using plant derivatives such as essential oils, their fractions, or isolated chemical components, it is necessary to broaden the knowledge about the use of these products currently available on the market (44).

Nowadays, researching repellents presents a challenge for several reasons, such as the limited knowledge of the physiological mechanisms that produce the repellent effect, the difficulty in testing and quantifying repellency, and the associated costs, among other variables. It is estimated that it can take about 10 years to bring a new insect-repellent product to market (23).

Among the difficulties encountered are the tests required to ensure the safety of the active ingredients. These include tests for acute toxicity, mucosal irritation, skin irritation, sensitization, toxicity, phototoxicity, photosensitization, photomutagenicity, absorption (*in vitro*/*in vivo*), teratogenicity, and others, when applicable (41).

In addition, despite all the advantages of using repellent products based on essential oils, there are limitations, such as their rapid volatilization and short action period. On the other hand, caution has been recommended in the use of essential oils due to potential toxic effects, including allergenicity, mutagenicity, and genotoxicity, which have been reported (41, 45). However, other studies have also tested repellents and insecticides derived from different plant essential oils and their components using a variety of methods, showing no mutagenic effects. Accordingly, the EPA has registered citronella essential oil, eucalyptus essential oil, and others as safe and effective ingredients for use in topical insect repellents (19). Therefore, despite the inherent repellent potential of essential oils, manufacturers often combine them with synthetic additives and isolated natural compounds to increase their effectiveness (41). This underscores that the development and commercialization of new repellents is a promising field requiring increased attention from researchers and manufacturers worldwide.

## Conclusions

The increasing spread of *Aedes aegypti* to new regions, driven by climate change and other factors, underscores the urgency of developing and promoting effective insect repellents as a key dengue prevention measure. While synthetic repellents such as DEET have dominated the market for decades, the demand for natural alternatives is rising due to their safety profiles and environmental friendliness. However, these products face limitations that must be addressed through improved formulations to increase the duration and effectiveness of protection.

It is essential that researchers and manufacturers focus their efforts on overcoming these challenges to develop more efficient and affordable repellents, particularly in regions with high dengue prevalence. This will not only contribute to the protection of at-risk populations but will also strengthen global vector-borne disease control strategies. Prevention policies, such as using repellents, are critical in the fight against the spread of dengue.

## Future directions

Dengue is spread worldwide through its vector *Aedes aegypti*, and for this reason, insect repellents are a significant tool to help control virus transmission. Nevertheless, only a handful of molecules have been scientifically validated for their activity. Therefore, there is an urgent need to actively search for synthetic, possibly rationally designed, mosquito-repellent molecules. In addition, a plethora of natural products contains millions of potential mosquito repellents that may be both safe and effective. This review invites investigators to explore this research opportunity, which has a significant impact on public health.
